# Participatory Monitoring and Evaluation of the COVID-19 Response in a Local Public Health System

**DOI:** 10.1177/15248399211041085

**Published:** 2021-09-30

**Authors:** Christina M. Holt, Stephen B. Fawcett, Ruaa Hassaballa-Muhammad, Dan Partridge, Sonia Jordan

**Affiliations:** 1 University of Kansas, Lawrence, KS, USA; 2 Lawrence–Douglas County Public Health, Lawrence, KS, USA

**Keywords:** COVID-19, pandemic response, county health departments, pandemic monitoring, participatory evaluation, monitoring and evaluation, collaborative action, community research, participatory research

## Abstract

The coronavirus disease 2019 (COVID-19) pandemic tested the capacity of local health systems to understand and respond to changing conditions. Although data on new cases of COVID-19 were widely shared in communities, there was less information on the multisector response activities and factors associated with implementation. To address this gap, this empirical case study examined (a) the pattern of implementation of COVID-19 response activities and (b) the factors and critical events associated with both the pattern of new cases and the implementation of the local COVID-19 response. We used a participatory monitoring and evaluation system to capture, code, characterize, and communicate 580 COVID-19 response activities implemented in the city of Lawrence and Douglas County, Kansas. Collaboration across sectors including public health, medical services, city/county government, businesses, social services, public schools, and universities enabled the local public health system’s response effort. Documentation results showed the varying pattern of new COVID-19 cases and response activities over time and the factors identified as enabling or impeding the response and related new cases. Similar participatory monitoring and evaluation methods can be used by local health systems to help understand and respond to the changing conditions of COVID-19 response and recovery.

The coronavirus disease 2019 (COVID-19) pandemic swept the world in 2020 (Alwan et al., 2020), with a highly varied impact on cases and deaths in different places (USA Facts, 2020; World Health Organization [WHO], 2020a). The public health toll—and related inequities—reflected different exposures, vulnerabilities, and willingness to take protections and governmental actions Lancet COVID-19 Commissioners, 2020; Schulz et al., 2020).

In February 2020, the WHO (2020b) outlined a roadmap for the COVID-19 response to limit transmission. The U.S. Centers for Disease Control and Prevention (CDC) also communicated technical guidance for COVID-19 responses: (a) surveillance and epidemiology, (b) risk communication and community engagement, (c) infection prevention and control, (d) case management and health services, and (e) laboratory services (CDC, 2020b, 2020c).

Locally and globally, multisectoral partnerships emerged to plan and implement responses to the pandemic. Surveillance systems yielded widely available information on patterns observed with new cases of COVID-19. However, systematic documentation of response activities was rare. This made it difficult to estimate the “dose” of the response and the factors that enabled or impeded efforts and was a barrier to making needed adjustments.

Although research can ultimately help discover what combinations of strategies are effective in mitigation (Hsiang et al., 2020), there is wide variation in the COVID-19 response in local communities. This empirical case study had two primary aims: (a) to capture and communicate COVID-19 response activities in a local public health system and (b) to facilitate participatory sense making in which partners identify factors related to new cases and response activities.

## Method

### Local Context and Collaborating Partners

This study examined the COVID-19 response as implemented in Lawrence, Kansas, and surrounding Douglas County (total population 122,259). From March 1 to November 30, 2020 (the study period), there were 4,880 new cases of COVID-19, with 25 associated deaths.

Lawrence–Douglas County Public Health coordinated the local public health system’s COVID-19 response. The University of Kansas (KU) Center, as part of its academic health department relationship with Lawrence–Douglas County Public Health and with support from the Kansas Health Foundation, designed and implemented the monitoring and evaluation (M&E) system for the public health system’s COVID-19 response.

The KU team brought experience in monitoring and evaluating the Ebola response in Liberia, in collaboration with the World Health Organization Regional Office for Africa (Hassaballa et al., 2019; Munodawafa et al., 2018; Sepers et al., 2018). The KU team also had concurrent experience partnering with the WHO Africa Regional Office designing and implementing a similar M&E system for examining the COVID-19 response in 47 countries in Africa.

### Conceptual Framework and Intervention Components of the Local COVID-19 Response

Consistent with the Institute of Medicine’s framework for collaborative public health action in communities (Fawcett et al., 2016; Institute of Medicine, 2003), the local pandemic response included phases of (a) assessment, (b) planning, (c) implementing targeted action, (d) changing conditions and systems, (e) achieving widespread change in behavior, and (f) improving population-level outcomes.

Response efforts in Lawrence–Douglas County consisted of eight components: (a) surveillance and epidemiology (e.g., collecting information about new cases of COVID-19 from health providers), (b) risk communication and community engagement (e.g., communicating information about mask wearing and handwashing), (c) infection prevention and control (e.g., stay-at-home orders; bar closings), (d) case management and health services (e.g., contact tracing to find and isolate those exposed to infected patients), (e) laboratory services (e.g., testing and reporting results of clinical samples for COVID-19), (f) supply procurement and logistics (e.g., obtaining and distributing needed supplies of personal protective equipment), (g) coordination (e.g., forming structures and joint plans for responding to COVID-19), and (h) maintenance of essential services and operations (e.g., primary care, social services for those experiencing homelessness).

Table 1 provides an overview of the local COVID-19 response intervention components, illustrative response activities implemented, and the sectors involved.

**Table 1:** Local COVID-19 Response Intervention Components, Illustrative Response Activities Implemented, and Sectors Involved in Delivery

*Response intervention components*	*Illustrative response activities implemented*	*Sectors involved in delivery*
Surveillance and epidemiology	Contact tracing and epidemiology investigations implemented	Public health, county government
Risk communication and community engagement	Unified Command launched the “Smart & Safe Community COVID-19 Scorecard,” a guiding document for decision making	Hospital, public health department, education/schools and universities, city government, county government
Infection prevention and control	Public health order required establishments to stop serving alcohol by 9 p.m. and close by 10 p.m.	City government, county government, public health, business, law enforcement
Case management and health services	Nurses visited the local homeless shelter two times per week to provide COVID-19 screening and education	Public health, social service
Laboratory services	The university expanded symptomatic testing capacity with a drive-through clinic in a dorm parking lot	Health providers, hospital, university
Supply procurement and logistics	Hospitals partnered with health centers on supply procurement for needed swabs	Health providers, hospital
Coordination	Unified Command activated under the National Incident Management System	Public health, emergency management, hospital, city and county government, schools, universities, law enforcement, health care providers, human service providers, businesses
Maintenance of essential services and operations	County Commission provided additional funding to local food banks to support increase in food insecurity	Local government, social service

*Note*. COVID-19 = coronavirus disease 2019.

### Evaluation Questions and Participatory M&E Approach

This study examined two evaluation questions (CDC, 2020a) related to mitigation of COVID-19 in the local community: (a) “What factors or critical events were associated with increases and decreases in the pattern of new cases of COVID-19 in the community?” and (b) “What factors or critical events were identified as enabling or impeding the COVID-19 response of the local public health system?”

A participatory M&E system (Center for Community Health and Development, n.d.; Fawcett et al., 2015; Hassaballa et al., 2019) was used to capture, code, characterize, and communicate the COVID-19 response by the local public health system. The team captured COVID-19 response activities using interviews with key actors in relevant sectors (e.g., public health leadership, director of emergency preparedness, city managers, county administrators, hospital administration, federally qualified health care centers, lead businesses, schools, social services, and university staff). The KU Center team also gathered and reviewed documents, including activity logs, minutes of Unified Command and school board meetings, and press releases for response activities.

As part of this participatory evaluation approach, KU Center staff also facilitated a series of four sense-making sessions to support review of the data. These sessions were typically scheduled adjacent to other meetings to be sensitive to partners’ time demands and lasted for 45 minutes to 1 hour. Participants ranged from six to nine in each session and included subsets of leadership from the Lawrence–Douglas County Public Health and Unified Command (e.g., city manager, county administrator, head of chamber of commerce, emergency operations staff, school and university administrators, local hospital staff, communications staff, data analysts, and a clinic director). These sense-making sessions prompted dialogue on (a) patterns seen in graphs of new cases and response activities, (b) candidate factors and critical events identified as affecting new cases and to enabling or impeding the response, (c) additional data partners would like to review, and (d) lessons learned and areas for adjustment.

#### Measurement

The COVID-19 response M&E system supported capturing response activities and communicating what partners identified as important in the response. Partners used the M&E system (Fawcett & Schultz, 2008) to (a) *capture* COVID-19 response activities (i.e., *who* implemented the activity/change, *what* they did, toward *what goal*, *with whom*, and *how many* were affected), (b) *code* activities using established definitions and scoring instructions, (c) *characterize* attributes of activities (e.g., type of COVID-19 responses addressed, sectors involved, what the vulnerable population was intended to benefit), and (d) *communicate* findings through graphs, sense-making dialogues, presentations, and reports.

The four types of COVID-19 response activities coded included (a) *community/system changes*: new or modified programs, policies, and practices implemented to reduce transmission of COVID-19 or respond to community needs (e.g., policy changes such as stay-at-home order or bar closings; housing residents experiencing homelessness to be transferred to hotel rooms to ensure shelter and adequate distancing); (b) *developmental activities*: actions taken to enable the group to reach its goals (e.g., developing assessment protocols, strategic plans, public communication plans); (c) *services provided*: delivery of information, training, or other valued goods (e.g., testing; food assistance); and (d) *resources generated*: acquisition of resources through grants, donations, or gifts in kind (e.g., federal funding, donated masks).

To assure data accuracy, primary observers and a secondary observer used definitions and scoring instructions for key activities to independently code documented activities (*N* = 580 entries). Interobserver reliability was computed by dividing the number of entries coded identically by both observers (*N* = 507) by the number of entries both documenters coded (*N* = 513). Interobserver agreement for documented activities was 98.8%, providing some assurance that scoring of discrete instances of documented response activities was accurate and reliable.

As part of surveillance activities, the Lawrence–Douglas County Public Health team gathered and communicated data on 14-day rolling averages of new COVID-19 cases. This made it possible to integrate quantitative data on new cases and response activities with qualitative data on identified factors associated with increases/decreases from sense-making sessions with leadership of the Unified Command structure.

#### Case Study Design

This empirical case study design (Yin, 2013) examines patterns in new cases and COVID-19 response activities as well as factors identified as contributing to changes in observed patterns.

## Results

### What Factors or Critical Events Were Associated With Increases or Decreases in the Pattern of New Cases of COVID-19 in the Community?

**Figure 1:**
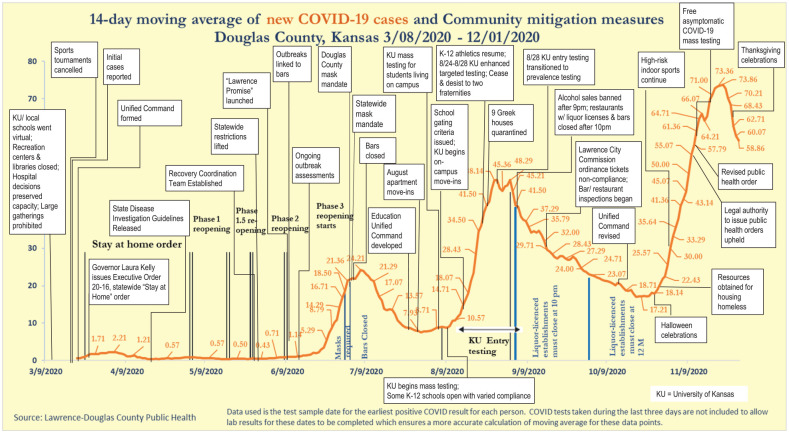
The 14-Day Moving Average of New COVID-19 Cases in Douglas County, Kansas, and Associated Factors Identified by Local Pandemic Partners

Figure 1 shows the 14-day moving average of new COVID-19 cases in Douglas County and the associated factors or critical events identified by local partners.

Candidate factors identified by partners are represented by text boxes overlaid on the graph. The pattern shows a low level of new cases from March until mid-June 2020, followed by different marked rises and gradual falls in reported cases, with cases showing a series of peaks in July, September, and November of the study period.

Candidate factors related to the pattern of new cases were identified during sense-making sessions with local partners. Key events or factors associated with delaying the rise in initial cases included local school and university decisions to hold classes online following spring break, closing recreation centers and libraries, a statewide stay-at-home order, and prohibition of large gatherings. Following the first recorded COVID-19 case, key events identified for infection prevention and control included forming a Unified Command structure for COVID-19 response, state and local stay-at-home orders, and changes in business practices. Factors associated with the first marked rise in cases included lifting of statewide restrictions, outbreaks in bars, and fuller reopening of businesses. Factors associated with bending the curve following the first rise included Douglas County and statewide mask-wearing mandates and bar closings.

Factors associated with a second marked rise in new cases included KU students moving back to town and into congregate housing and KU mass testing for students, faculty, and staff. Key events associated with a reduction in cases following this second rise included KU cracking down on fraternities not complying with COVID safety, banning alcohol sales in later hours, bar/restaurant inspections, and a Lawrence City Commission ordinance to ticket COVID noncompliance. KU reduced its testing to a more targeted approach later in August, so this may have led to reduction in detection of cases. Ongoing factors include some K–12 schools opening, with varied compliance with public health guidance (e.g., allowing fall/winter sports). Factors associated with increased cases from September and continuing through November included social gatherings, athletics, and inability to socially distance in congregate living settings.

### What Factors or Critical Events Were Identified as Enabling or Impeding the COVID-19 Response in the Local Public Health System?

**Figure 2:**
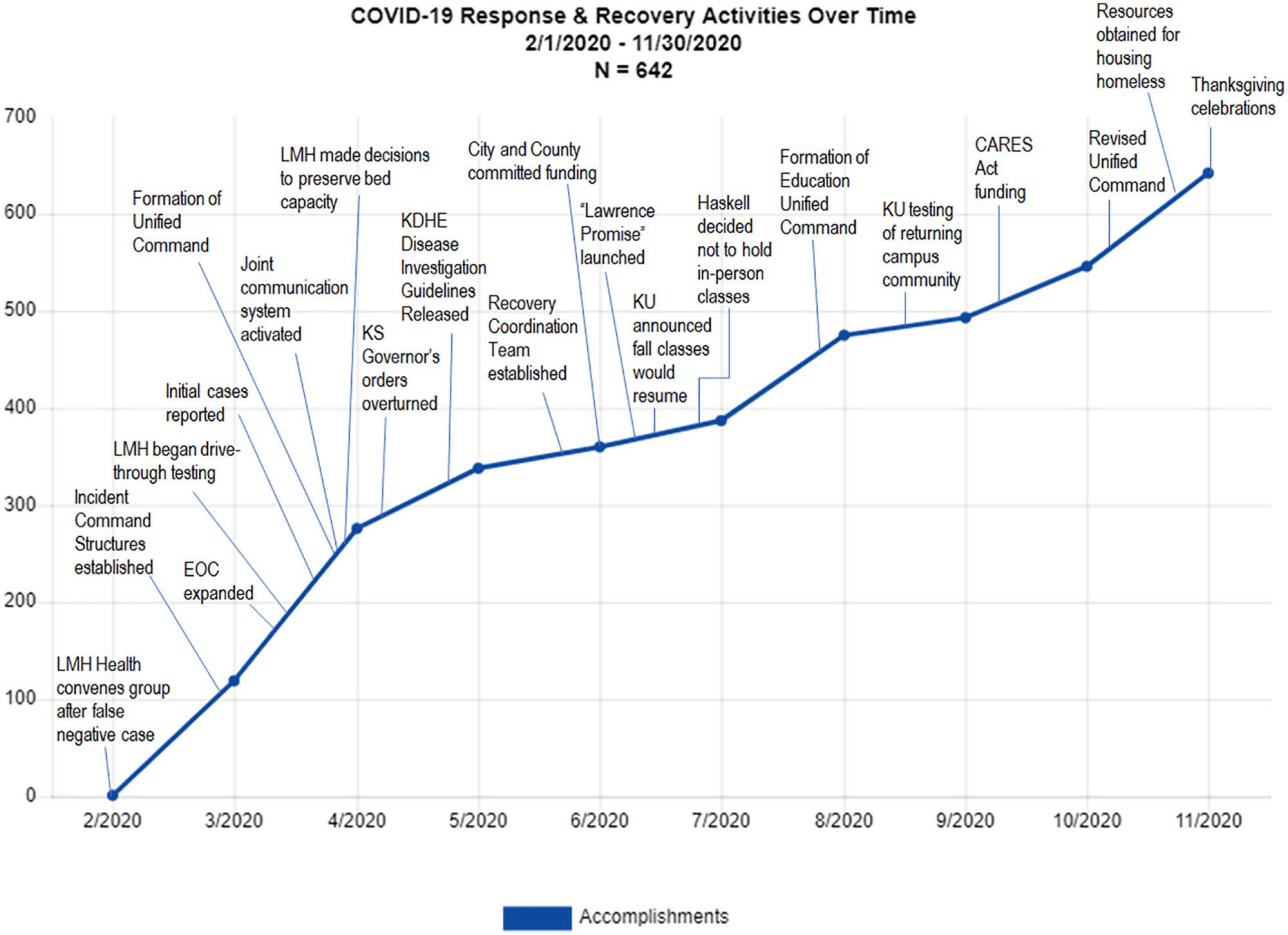
Cumulative Number of COVID-19 Response and Recovery Activities Over Time and Factors Identified by Partners as Enabling Implementation in Douglas County, Kansas

Figure 2 shows COVID-19 response activities implemented in Lawrence–Douglas County. These were related to the COVID-19 response effort’s eight components (e.g., surveillance and epidemiology, risk communication, infection prevention and control, case management and health services). See Table 1 for illustrative response activities.

From February 2020 through November 2020 (the study period), there were 642 COVID-19 response activities documented in the Douglas County. This graph displays a cumulative record of the unfolding of activities captured in the M&E system, with each new activity added to all previous activities. The steeper the line, the higher the rate of response and recovery activities implemented. Activities began in early February 2020 in response to global and national reports of the pandemic before the first identified local case in the third week of March. An initial burst of activity (steeper line) was seen after the incident command structures were established through May, with a steady pattern of response activity observed throughout the study period and an increase in activity following the CARES (Coronavirus Aid, Relief, and Economic Security Act) Act funding and the revision of the Unified Command structure.

Candidate factors related to implementation (shown as text boxes, timed to onset) were identified during sense-making sessions with the Lawrence–Douglas County Public Health leadership and the leadership of the Unified Command. Key events and factors associated with the initial response activities (even before the first local case) included convening of partners after a false-negative case and establishing the incident command structure. Other factors included existing collaborative relationships, earlier adoption and implementation of the National Incident Management System, joint communication efforts, establishing recovery coordination teams, expanded testing, KU/school decisions to reopen for the fall semester, early commitments of city/county funding for response activities, and a reconstitution of the Unified Command.

Table 2 summarizes factors identified by partners as enabling or impeding local public health system COVID-19 response activities. Enabling factors included the local public health department’s size, experience, and capacity; existing collaborative relationships; establishing a multisector Unified Command structure to strengthen communication and coordination; and political support from city and county commissioners for creating and enforcing public health orders.

**Table 2:** Factors Identified by Partners as Enabling or Impeding Local COVID-19 Response Activities

*Enabling factors*: • Local public health department size, experience, and capacity to adapt and do the work • Existing collaborative relationships among partners enabled trust and sharing of resources and responsibilities for addressing the pandemic • Establishing a Unified Command structure to strengthen communication and coordination across sectors (e.g., public health, hospital, education, business, human services) • Identification of equity advisors to serve on each Unified Command working group • Joint risk communication efforts to promote mask wearing, social distancing, and so on • Expanding testing (e.g., drive-through testing, testing for university students, asymptomatic mass testing) • Disease investigation guidance from state health department/expanded contact tracing • Adoption and implementation of the National Incident Management System • Establishing recovery coordination teams • Early commitments of city/county government funding for response activities • Receiving CARES Act funding • Early school decisions to reopen for the fall semester–enabled planning • Political support from city and county commissioners for creating and enforcing public health orders
*Impeding factors*: • Kansas legislature limiting the governor’s pandemic response power and placing local limitations on contact tracing (House Bill, 2016) • Pressure (social, political) to allow gatherings in public places and athletic events • Lawsuits against the local health department/city/county for restrictions placed on bars • Refusal by some residents to halt social gatherings, such as birthday parties and house parties • “COVID fatigue” and prematurely easing up on precautions • Difficulty building relationships and resolving conflicts due to limited in-person contact • Increased and competing demands on staff (e.g., to prepare for new activities, resist opposition, hire and onboard new staff, respond to questions from the public) • Prolonged stress on staff responsible for managing the pandemic response • Ambiguity about future funding to support response activities

*Note*. COVID-19 = coronavirus disease 2019.

Factors identified as impeding the response included the Kansas legislature limiting the governor’s pandemic response power, ambiguity about availability of future funding, pressure to allow public gatherings and athletics, prolonged stress on pandemic response staff, and lawsuits against the local health department for restrictions placed on bars.

## Discussion

This empirical case study had two primary aims: (a) to capture and communicate COVID-19 response activities in a local public health system and (b) to facilitate participatory sense making for partners to identify factors related to both new cases and the response effort. To address the first aim, in partnership with Lawrence–Douglas County Public Health, the KU team designed and implemented a customized M&E system (Fawcett & Schultz, 2008) to capture and communicate the local COVID-19 response. The M&E system enabled availability of real-time data for local partners and was tailored to capture and characterize key aspects of the effort, including (a) the type of COVID-19 response addressed (e.g., surveillance, risk communication and community engagement, infection prevention and control), (b) the aspect of recovery (e.g., economic recovery, housing and human services, social and emotional health), (c) the sector in which implemented (e.g., health, housing, education, business, city/county government), and (d) the vulnerable population intended to benefit (e.g., older adults, health workers, business workers, those experiencing homelessness).

To address the second aim, the KU team engaged local partners in systematically reflecting on what they were seeing in the data, what it meant, and implications for adjustment. Dialogue helped identify candidate factors associated with (a) the changes in new cases (e.g., “What factors or key events may have led to increases/decreases in news cases?”) and (b) the changes in the level of response activity (e.g., “What conditions or factors enabled/impeded response activities?”). These facilitated dialogues supported partners in identifying candidate factors affecting new cases and the response. This integration of quantitative and qualitative information yielded recommendations for practice that optimize enabling factors (e.g., establish command structures) and respond to impeding factors (e.g., assure social supports for stress management among staff).

A single case study design, such as this one, cannot yield evidence of a causal relationship between candidate factors—singly, or in combination—and changes in levels of either new cases or response activity. Rather it can identify candidate factors associated with changes in patterns that may be worthy of further testing in practice. The validity of these candidate factors is strengthened, however, by a participatory M&E approach in which potentially influencing conditions and interventions were identified by partners with deep experience in the local context.

## Implications For Practice

The data from this case study suggest the importance of establishing collaborative relationships with community partners well in advance of a crisis. Trusted partnerships lay the foundation for expedited planning and action. Additionally, it is important to take care to create an organizational structure that can assure effective communication and coordinated response efforts. The support of local government enhanced, and prolonged the implementation of, preventive measures enacted to protect the public’s health.

Participatory M&E enables partners to capture, characterize, and communicate their collaborative action. Stakeholders’ engagement in systematic reflection on the data shares power in determining the meaning of the data and its implications for needed adjustments. Participatory approaches to M&E can help generate practice guidance to plan, adapt, and guide collaborative action. Such practical knowledge—when informed by community engagement—can help strengthen efforts to address the ever-changing conditions of disease outbreaks and other challenges to community health.
